# Aberdeen and Its Institutions

**Published:** 1894-03-17

**Authors:** 


### ABERDEEN AND ITS INSTITUTIONS.

THE INFIRMARY BUILDINGS.

The transformation of the Royal Aberdeen Infirmary into a model hospital is being rapidly proceeded with. It will be remembered that the reconstruction of the present building on entirely new lines has been gradually progressing for some time, and the last stage of the work is now within measurable distance. It is very clear that the authorities have been all along animated with a spirit of practical common sense, which is a guarantee that the result will be excellent, and it would be well if the words used by Mr. Littlejohn, the chairman of the board of directors, at the annual meeting the other day, found echo among all those who are responsible for the erection and maintenance of charitable institutions. In stating the financial position of the infirmary at the present time, and explaining the existing condition of affairs with regard to the new buildings, Mr. Littlejohn said: "Our guiding principle has been that we should construct the best hospital that we possibly could, a hospital of the first class, a hospital which the people of Aberdeen will be proud of, and which they will be glad that strangers from any part of the country should come and see. It has been our leading principle, and while we have endeavoured to be economical in all points where economy would not affect the efficiency of the institution, we have never been frightened at any outlay which was calculated to enhance the value and usefulness of the institution." Would that all new buildings and extensions were planned on such eminently wise lines!

The work of the infirmary has been carried on under most trying circumstances during the past year while the process of converting the front portion of the hospital into an administration block has been proceeding, and only those intimately aware of hospital routine can perhaps fully realise exactly how trying such times can be. It had been hoped that the remodelling of the administration block would have been by this further advanced, but rapid progress has been impossible owing to the complicated nature of the work to be done. And during this protracted period the nursing staff have been houseless, so far as the infirmary itself is concerned, and have had the very considerable addition to their arduous work of daily walks to and from their temporary quarters outside the building. Half of the entire hospital has been in the hands of the workpeople, a sufficient indication of the many trials consequent upon such a condition of affairs.

### An Appeal for Increased Funds.

A sum of £20,000 is required to complete the buildings, which the general meeting of managers has given the board of directors power to borrow. With the enlargement and improvement of the infirmary an increase in annual expenditure will also have to be met, and Mr. Littlejohn's appeal to the people of Aberdeen for this purpose ought to meet with a hearty response. A little increased enthusiasm will do wonders, and indeed in several instances already where warm interest has been excited in certain districts the result has been most encouraging. With a view to further increase the income it is proposed to raise the present scale of students' fees, which are now quite inadequate, and much below those paid in other institutions, to a more proportionate sum. In asking for a greater degree of support to enable the infirmary to carry on its good work well and efficiently, the Chairman made a suggestion which may well be taken to heart by the wealthy citizens of other places than Aberdeen. Mr. Littlejohn called upon those with abundant means to apply a stricter revision to their scale of giving for the benefit of the sick poor, and a more serious consideration to such claims as against the growing expenditure upon personal luxuries now so general. Such an exhortation should bear good fruit in a largely increased support of the splendid medical institutions of which Aberdeen may be justly proud, and should be prouder yet in days to come.

### The Nursing Department.

The Royal Infirmary owes an immense debt of gratitude to its Honorary Superintendent, Miss Lumsden, whose unwearied labours for many years past have done much to raise the standard of nursing to its present high level. Her unselfish devotion is justly appreciated by the directors, who can find no language strong enough to express their sense of the value of her services.

The accommodation for the nursing staff in the new building, when complete, will be admirable in every respect, and should leave nothing to be desired in the matter of comfort.

In addition to the special field of work inside the infirmary walls, the management have been able during the past year to furnish skilled nursing help in the city and district sufficient to meet the demand, and have thus conferred a most valuable service to many.

### The Convalescent Hospital.

The convalescent hospital in connection with the infirmary has steadily carried on its useful work, but is sadly crippled by want of funds, the income for 1893 falling considerably below the expenditure, leaving a debt on the institution of £474. It has been decided to dispose of the present premises to the asylum board of directors to the mutual advantage of both institutions, the present site being unsuitable for the hospital, while its acquisition by the asylum authorities will be of great value. A stipulation has been made for a lease of two years at a rent of £65, this arrangement giving ample time for the securing of appropriate quarters elsewhere. The sum to be paid for the property has been fixed at £3,800.

This year sees for the first time the uniform system of accounts adopted by the Aberdeen Infirmary authorities.

### The Royal Asylum.

A steady increase in the number of patients has unfortunately to be chronicled of recent years. Under the admirable management of Dr. Reid the inmates of this asylum received every possible advantage of treatment and care, and systematic instruction is now being given to the attendants and nurses by Dr. Angus, the senior assistant medical officer, in order that they may be qualified to compete for the certificate granted to asylum nurses and attendants by the Medico-Psychological Association of Great Britain and Ireland.

A suggestion has been made by Dr. Reid as to the propriety of changing the name of the institution from that of Asylum, with its prison-like associations, to that of "Hospital for Diseases of the Nervous System," or some similar title—a wise proposal, which it is to be hoped may be carried out. The acquisition of the convalescent hospital, as already pointed out, will be a considerable improvement. The Daviot branch of the institution is also undergoing substantial improvements, and great interest is manifested in the work by the patients, to whom it has been of material benefit.

